# Oxomemazine hydro­chloride

**DOI:** 10.1107/S1600536811025372

**Published:** 2011-07-02

**Authors:** M. S. Siddegowda, Ray J. Butcher, Mehmet Akkurt, H. S. Yathirajan, A. R. Ramesh

**Affiliations:** a Department of Studies in Chemistry, University of Mysore, Manasagangotri, Mysore 570 006, India; b Department of Chemistry, Howard University, 525 College Street NW, Washington, DC 20059, USA; c Department of Physics, Faculty of Sciences, Erciyes University, 38039 Kayseri, Turkey; d R. L. Fine Chem, Bangalore 560 064, India

## Abstract

In the title compound [systematic name: 3-(5,5-dioxo­phen­othia­zin-10-yl)-*N*,*N*,2-trimethyl­propanaminium chloride], C_18_H_23_N_2_O_2_S^+^·Cl^−^, the dihedral angle between the two outer aromatic rings of the phenothia­zine unit is 30.5 (2)°. In the crystal, the components are linked by N—H⋯Cl and C—H⋯Cl hydrogen bonds and C—H⋯π inter­actions.

## Related literature

For background to oxomemazine, see: Amin *et al.* (2008 ▶); El-Didamony, (2005 ▶). For related structures, see: Harrison *et al.* (2007 ▶); Jasinski *et al.* (2011 ▶).
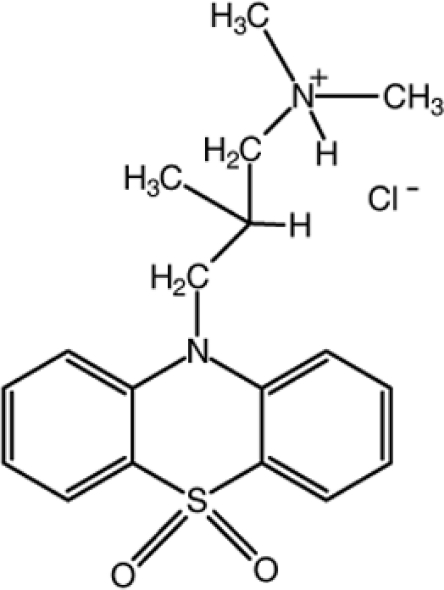


## Experimental

### 

#### Crystal data

C_18_H_23_N_2_O_2_S^+^·Cl^−^
*M* *_r_* = 366.90
Triclinic, 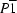

*a* = 7.6364 (7) Å
*b* = 10.4177 (9) Å
*c* = 12.4732 (10) Å
α = 103.478 (7)°
β = 90.624 (7)°
γ = 109.852 (8)°
*V* = 903.21 (15) Å^3^
*Z* = 2
Cu *K*α radiation
μ = 3.06 mm^−1^
*T* = 295 K
0.32 × 0.25 × 0.24 mm

#### Data collection

Oxford Diffraction Xcalibur Ruby Gemini diffractometer
Absorption correction: refined from Δ*F* [*XABS2* (Parkin *et al.*, 1995 ▶) in *WinGX* (Farrugia (1999 ▶)]*T*_min_ = 0.441, *T*_max_ = 0.528
6466 measured reflections
3598 independent reflections
3120 reflections with *I* > 2σ(*I*)
*R*_int_ = 0.0422

#### Refinement

*R*[*F* ^2^ > 2σ(*F* ^2^)] = 0.079
*wR*(*F* ^2^) = 0.229
*S* = 1.09
3598 reflections
221 parameters
H-atom parameters constrained
Δρ_max_ = 0.48 e Å^−3^
Δρ_min_ = −0.55 e Å^−3^

### 

Data collection: *CrysAlis PRO* (Oxford Diffraction, 2007 ▶); cell refinement: *CrysAlis PRO*; data reduction: *CrysAlis RED* (Oxford Diffraction, 2007 ▶); program(s) used to solve structure: *SHELXS97* (Sheldrick, 2008 ▶); program(s) used to refine structure: *SHELXL97* (Sheldrick, 2008 ▶); molecular graphics: *ORTEP-3 for Windows* (Farrugia, 1997 ▶); software used to prepare material for publication: *WinGX* (Farrugia, 1999 ▶) and *PLATON* (Spek, 2009 ▶).

## Supplementary Material

Crystal structure: contains datablock(s) global, I. DOI: 10.1107/S1600536811025372/hb5925sup1.cif

Structure factors: contains datablock(s) I. DOI: 10.1107/S1600536811025372/hb5925Isup2.hkl

Supplementary material file. DOI: 10.1107/S1600536811025372/hb5925Isup3.cml

Additional supplementary materials:  crystallographic information; 3D view; checkCIF report

## Figures and Tables

**Table 1:** Hydrogen-bond geometry (Å, °) *Cg*2 is the centroid of the C1–C6 benzene ring.

*D*—H⋯*A*	*D*—H	H⋯*A*	*D*⋯*A*	*D*—H⋯*A*
N2—H2*B*⋯Cl1	0.91	2.18	3.027 (4)	155
C13—H13*A*⋯Cl1^i^	0.97	2.80	3.608 (4)	141
C13—H13*B*⋯Cl1	0.97	2.76	3.692 (4)	161
C17—H17*B*⋯*Cg*2^ii^	0.96	2.62	3.559 (6)	166

Symmetry codes: (i) 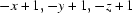; (ii) 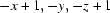.

**Fig. 1.**
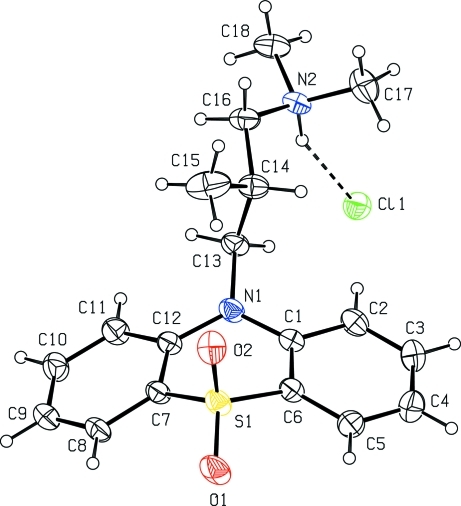
View of (I) showing displacement ellipsoids for non-H atoms drawn at the 30% probability level.

**Fig. 2.**
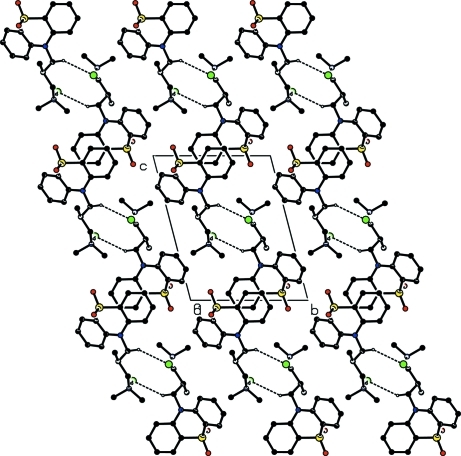
A view of the crystal packing and hydrogen bonding of (I) shown down the a axis.

## supplementary crystallographic information

### Comment

Oxomemazine is an antihistamine and anticholinergic of the phenothiazine chemical class used for the treatment of cough. The extractive spectrophotometric methods for the determination of oxomemazine hydrochloride in bulk and pharmaceutical formulations using some organic dyes is described (El-Didamony, 2005; Amin *et al.*, 2008). The crystal structures of dioxopromethazinium picrate (Harrison *et al.*, 2007) and 1-(10*H*-phenothiazin-2-yl)ethanone (Jasinski *et al.*, 2011) have been reported. We now report the crystal structure of the title compound, (I).

In the molecule of (I), (Fig. 1), the dihedral angle between the two aromatic rings of the phenothiazine unit is 30.5 (2)°. All bond lengths and angles in (I) are normal. In the crystal structure, N—H···Cl, C—H···Cl hydrogen bonds (Table 1, Fig. 2) and C—H···π interactions help to establish the packing of (I).

### Experimental

The title compound was obtained as a gift sample from *R. L*. Fine Chem., Bangalore, India. X-ray quality crystals were obtained from a 1:1 mixture of dimethylformamide and ethanol by slow evaporation (m.p.: 520–523 K).

### Refinement

All H atoms were located geometrically (methyl C—H = 0.98 Å, methylene C—H = 0.99 Å, aromatic C—H = 0.95Å and N—H = 0.91 Å) and refined using a riding model. Their isotropic displacement parameters were set to 1.2 (or 1.5 for the methyl group) times the U_eq_ of the parent atom. 23 poorly fitted reflections were omitted from the refinement.

### Crystal data

**Table:**

C_18_H_23_N_2_O_2_S^+^·Cl^−^	*Z* = 2
*M**_r_* = 366.90	*F*(000) = 388
Triclinic, *P*1	*D*_x_ = 1.349 Mg m^−^^3^
Hall symbol: -P 1	Cu *K*α radiation, λ = 1.54178 Å
*a* = 7.6364 (7) Å	Cell parameters from 3224 reflections
*b* = 10.4177 (9) Å	θ = 4.7–75.0°
*c* = 12.4732 (10) Å	µ = 3.06 mm^−^^1^
α = 103.478 (7)°	*T* = 295 K
β = 90.624 (7)°	Prism, colourless
γ = 109.852 (8)°	0.32 × 0.25 × 0.24 mm
*V* = 903.21 (15) Å^3^	

### Data collection

**Table:**

Oxford Diffraction Xcalibur Ruby Gemini diffractometer	3598 independent reflections
Radiation source: Enhance (Cu) X-ray Source	3120 reflections with *I* > 2σ(*I*)
graphite	*R*_int_ = 0.074
Detector resolution: 10.5081 pixels mm^-1^	θ_max_ = 75.8°, θ_min_ = 4.7°
ω scans	*h* = −9→9
Absorption correction: part of the refinement model (Δ*F*) [*XABS2* (Parkin *et al.*, 1995) in the *WinGX* (Farrugia (1999).	*k* = −12→12
*T*_min_ = 0.441, *T*_max_ = 0.528	*l* = 0→15
6466 measured reflections	

### Refinement

**Table:**

Refinement on *F*^2^	Primary atom site location: structure-invariant direct methods
Least-squares matrix: full	Secondary atom site location: difference Fourier map
*R*[*F*^2^ > 2σ(*F*^2^)] = 0.079	Hydrogen site location: inferred from neighbouring sites
*wR*(*F*^2^) = 0.229	H-atom parameters constrained
*S* = 1.09	*w* = 1/[σ^2^(*F*_o_^2^) + (0.1168*P*)^2^ + 1.2778*P*] where *P* = (*F*_o_^2^ + 2*F*_c_^2^)/3
3598 reflections	(Δ/σ)_max_ < 0.001
221 parameters	Δρ_max_ = 0.48 e Å^−^^3^
0 restraints	Δρ_min_ = −0.55 e Å^−^^3^

### Special details

**Table:**

Geometry. Bond distances, angles *etc*. have been calculated using the rounded fractional coordinates. All su's are estimated from the variances of the (full) variance-covariance matrix. The cell e.s.d.'s are taken into account in the estimation of distances, angles and torsion angles
Refinement. Refinement on *F*^2^ for ALL reflections except those flagged by the user for potential systematic errors. Weighted *R*-factors *wR* and all goodnesses of fit *S* are based on *F*^2^, conventional *R*-factors *R* are based on *F*, with *F* set to zero for negative *F*^2^. The observed criterion of *F*^2^ > σ(*F*^2^) is used only for calculating -*R*-factor-obs *etc*. and is not relevant to the choice of reflections for refinement. *R*-factors based on *F*^2^ are statistically about twice as large as those based on *F*, and *R*-factors based on ALL data will be even larger.

### Fractional atomic coordinates and isotropic or equivalent isotropic displacement parameters (Å^2^)

**Table:**

	*x*	*y*	*z*	*U*_iso_*/*U*_eq_	
S1	0.59166 (14)	0.20629 (10)	0.95014 (7)	0.0449 (3)	
O1	0.5426 (5)	0.1883 (4)	1.0580 (2)	0.0663 (10)	
O2	0.7225 (4)	0.1441 (3)	0.9009 (3)	0.0587 (10)	
N1	0.5526 (5)	0.3244 (3)	0.7585 (3)	0.0434 (9)	
N2	0.5857 (5)	0.2792 (4)	0.3894 (3)	0.0448 (10)	
C1	0.4037 (5)	0.2045 (4)	0.7654 (3)	0.0401 (10)	
C2	0.2542 (6)	0.1382 (4)	0.6801 (3)	0.0492 (11)	
C3	0.1031 (6)	0.0237 (5)	0.6921 (4)	0.0552 (14)	
C4	0.0937 (6)	−0.0284 (5)	0.7851 (4)	0.0590 (14)	
C5	0.2388 (6)	0.0333 (4)	0.8684 (4)	0.0511 (12)	
C6	0.3941 (5)	0.1471 (4)	0.8570 (3)	0.0412 (10)	
C7	0.6735 (5)	0.3847 (4)	0.9521 (3)	0.0412 (11)	
C8	0.7662 (6)	0.4839 (4)	1.0502 (3)	0.0495 (11)	
C9	0.8514 (6)	0.6230 (5)	1.0501 (4)	0.0547 (12)	
C10	0.8428 (6)	0.6621 (4)	0.9525 (4)	0.0540 (11)	
C11	0.7472 (6)	0.5662 (4)	0.8560 (4)	0.0503 (12)	
C12	0.6580 (5)	0.4229 (4)	0.8529 (3)	0.0400 (10)	
C13	0.5914 (6)	0.3516 (4)	0.6489 (3)	0.0443 (11)	
C14	0.6707 (6)	0.2476 (5)	0.5775 (3)	0.0494 (12)	
C15	0.8375 (9)	0.2381 (8)	0.6378 (5)	0.084 (2)	
C16	0.7363 (6)	0.2971 (5)	0.4743 (3)	0.0507 (14)	
C17	0.4856 (8)	0.1292 (5)	0.3288 (5)	0.0709 (17)	
C18	0.6669 (8)	0.3653 (6)	0.3094 (4)	0.0664 (16)	
Cl1	0.22428 (14)	0.32837 (11)	0.44260 (9)	0.0524 (3)	
H2A	0.25720	0.17110	0.61670	0.0590*	
H2B	0.50060	0.31300	0.42450	0.0540*	
H3A	0.00520	−0.01930	0.63590	0.0660*	
H4A	−0.00980	−0.10460	0.79140	0.0710*	
H5A	0.23360	−0.00030	0.93150	0.0610*	
H8A	0.77010	0.45550	1.11530	0.0600*	
H9A	0.91380	0.68960	1.11470	0.0650*	
H10A	0.90310	0.75580	0.95170	0.0650*	
H11A	0.74180	0.59690	0.79220	0.0600*	
H13A	0.68010	0.44670	0.65870	0.0530*	
H13B	0.47660	0.34550	0.61050	0.0530*	
H14A	0.57280	0.15410	0.55540	0.0590*	
H15A	0.79770	0.19790	0.69920	0.1250*	
H15B	0.92950	0.33080	0.66460	0.1250*	
H15C	0.89070	0.17960	0.58800	0.1250*	
H16A	0.81350	0.39620	0.49720	0.0610*	
H16B	0.81480	0.24610	0.44000	0.0610*	
H17A	0.41330	0.07910	0.37840	0.1060*	
H17B	0.57500	0.08670	0.30100	0.1060*	
H17C	0.40390	0.12520	0.26810	0.1060*	
H18A	0.72400	0.46260	0.34880	0.0990*	
H18B	0.56930	0.35570	0.25570	0.0990*	
H18C	0.75940	0.33290	0.27250	0.0990*	

### Atomic displacement parameters (Å^2^)

**Table:**

	*U*^11^	*U*^22^	*U*^33^	*U*^12^	*U*^13^	*U*^23^
S1	0.0587 (6)	0.0433 (5)	0.0309 (5)	0.0145 (4)	−0.0028 (4)	0.0112 (3)
O1	0.095 (2)	0.0633 (19)	0.0316 (15)	0.0125 (17)	−0.0023 (15)	0.0187 (13)
O2	0.0562 (17)	0.0536 (17)	0.069 (2)	0.0230 (14)	−0.0042 (15)	0.0155 (15)
N1	0.0551 (18)	0.0425 (16)	0.0297 (15)	0.0128 (14)	0.0014 (13)	0.0101 (12)
N2	0.0564 (18)	0.0517 (18)	0.0292 (15)	0.0247 (15)	0.0070 (13)	0.0070 (12)
C1	0.0470 (19)	0.0416 (18)	0.0321 (17)	0.0179 (15)	0.0046 (14)	0.0063 (14)
C2	0.058 (2)	0.053 (2)	0.0380 (19)	0.0230 (18)	−0.0036 (17)	0.0090 (16)
C3	0.048 (2)	0.051 (2)	0.058 (3)	0.0115 (17)	−0.0092 (18)	0.0070 (19)
C4	0.052 (2)	0.050 (2)	0.068 (3)	0.0080 (18)	0.001 (2)	0.017 (2)
C5	0.056 (2)	0.047 (2)	0.048 (2)	0.0138 (17)	0.0061 (18)	0.0143 (17)
C6	0.0487 (19)	0.0388 (17)	0.0350 (18)	0.0151 (15)	0.0026 (14)	0.0075 (14)
C7	0.052 (2)	0.0384 (18)	0.0298 (17)	0.0134 (15)	0.0039 (14)	0.0059 (13)
C8	0.059 (2)	0.052 (2)	0.0287 (18)	0.0134 (18)	−0.0002 (16)	0.0028 (15)
C9	0.062 (2)	0.050 (2)	0.041 (2)	0.0147 (19)	0.0038 (18)	−0.0018 (17)
C10	0.062 (2)	0.0389 (19)	0.052 (2)	0.0098 (17)	0.0027 (19)	0.0066 (17)
C11	0.062 (2)	0.044 (2)	0.044 (2)	0.0167 (18)	0.0038 (17)	0.0124 (16)
C12	0.0455 (18)	0.0410 (18)	0.0327 (17)	0.0156 (15)	0.0041 (14)	0.0074 (14)
C13	0.058 (2)	0.048 (2)	0.0305 (17)	0.0195 (17)	0.0049 (15)	0.0152 (15)
C14	0.057 (2)	0.061 (2)	0.040 (2)	0.0286 (19)	0.0092 (17)	0.0195 (18)
C15	0.082 (3)	0.145 (6)	0.068 (3)	0.072 (4)	0.026 (3)	0.060 (4)
C16	0.052 (2)	0.071 (3)	0.037 (2)	0.029 (2)	0.0118 (16)	0.0172 (18)
C17	0.080 (3)	0.054 (3)	0.067 (3)	0.022 (2)	−0.006 (3)	−0.004 (2)
C18	0.086 (3)	0.084 (3)	0.040 (2)	0.036 (3)	0.020 (2)	0.026 (2)
Cl1	0.0493 (5)	0.0565 (6)	0.0514 (6)	0.0189 (4)	0.0047 (4)	0.0133 (4)

### Geometric parameters (Å, °)

**Table:**

S1—O1	1.438 (3)	C14—C15	1.516 (9)
S1—O2	1.436 (3)	C14—C16	1.524 (6)
S1—C6	1.733 (4)	C2—H2A	0.9300
S1—C7	1.742 (4)	C3—H3A	0.9300
N1—C1	1.396 (5)	C4—H4A	0.9300
N1—C12	1.394 (5)	C5—H5A	0.9300
N1—C13	1.473 (5)	C8—H8A	0.9300
N2—C16	1.488 (6)	C9—H9A	0.9300
N2—C17	1.491 (7)	C10—H10A	0.9300
N2—C18	1.496 (7)	C11—H11A	0.9300
N2—H2B	0.9100	C13—H13A	0.9700
C1—C6	1.400 (5)	C13—H13B	0.9700
C1—C2	1.417 (6)	C14—H14A	0.9800
C2—C3	1.389 (7)	C15—H15A	0.9600
C3—C4	1.384 (7)	C15—H15B	0.9600
C4—C5	1.379 (7)	C15—H15C	0.9600
C5—C6	1.400 (6)	C16—H16A	0.9700
C7—C8	1.400 (5)	C16—H16B	0.9700
C7—C12	1.401 (5)	C17—H17A	0.9600
C8—C9	1.373 (6)	C17—H17B	0.9600
C9—C10	1.378 (7)	C17—H17C	0.9600
C10—C11	1.380 (7)	C18—H18A	0.9600
C11—C12	1.406 (6)	C18—H18B	0.9600
C13—C14	1.530 (6)	C18—H18C	0.9600
			
O1—S1—O2	117.1 (2)	C3—C4—H4A	120.00
O1—S1—C6	111.1 (2)	C5—C4—H4A	120.00
O1—S1—C7	110.2 (2)	C4—C5—H5A	120.00
O2—S1—C6	108.2 (2)	C6—C5—H5A	120.00
O2—S1—C7	108.99 (19)	C7—C8—H8A	120.00
C6—S1—C7	99.90 (19)	C9—C8—H8A	120.00
C1—N1—C12	121.7 (3)	C8—C9—H9A	121.00
C1—N1—C13	118.9 (3)	C10—C9—H9A	121.00
C12—N1—C13	119.3 (3)	C9—C10—H10A	119.00
C16—N2—C17	113.2 (4)	C11—C10—H10A	119.00
C16—N2—C18	109.4 (4)	C10—C11—H11A	119.00
C17—N2—C18	110.1 (4)	C12—C11—H11A	120.00
C16—N2—H2B	108.00	N1—C13—H13A	109.00
C17—N2—H2B	108.00	N1—C13—H13B	109.00
C18—N2—H2B	108.00	C14—C13—H13A	109.00
N1—C1—C2	120.7 (3)	C14—C13—H13B	109.00
N1—C1—C6	121.7 (4)	H13A—C13—H13B	108.00
C2—C1—C6	117.6 (4)	C13—C14—H14A	109.00
C1—C2—C3	119.4 (4)	C15—C14—H14A	109.00
C2—C3—C4	122.0 (4)	C16—C14—H14A	109.00
C3—C4—C5	119.6 (5)	C14—C15—H15A	109.00
C4—C5—C6	119.4 (4)	C14—C15—H15B	109.00
S1—C6—C1	118.3 (3)	C14—C15—H15C	110.00
S1—C6—C5	119.3 (3)	H15A—C15—H15B	109.00
C1—C6—C5	122.0 (4)	H15A—C15—H15C	109.00
C8—C7—C12	122.2 (4)	H15B—C15—H15C	110.00
S1—C7—C8	119.0 (3)	N2—C16—H16A	108.00
S1—C7—C12	118.6 (3)	N2—C16—H16B	108.00
C7—C8—C9	119.9 (4)	C14—C16—H16A	108.00
C8—C9—C10	118.8 (4)	C14—C16—H16B	108.00
C9—C10—C11	121.9 (4)	H16A—C16—H16B	107.00
C10—C11—C12	120.9 (4)	N2—C17—H17A	109.00
N1—C12—C7	121.2 (4)	N2—C17—H17B	110.00
N1—C12—C11	122.5 (4)	N2—C17—H17C	110.00
C7—C12—C11	116.2 (4)	H17A—C17—H17B	109.00
N1—C13—C14	112.6 (3)	H17A—C17—H17C	109.00
C13—C14—C15	112.2 (4)	H17B—C17—H17C	109.00
C13—C14—C16	109.1 (4)	N2—C18—H18A	109.00
C15—C14—C16	107.5 (4)	N2—C18—H18B	109.00
N2—C16—C14	115.8 (4)	N2—C18—H18C	109.00
C1—C2—H2A	120.00	H18A—C18—H18B	109.00
C3—C2—H2A	120.00	H18A—C18—H18C	110.00
C2—C3—H3A	119.00	H18B—C18—H18C	110.00
C4—C3—H3A	119.00		
			
O1—S1—C6—C1	154.8 (3)	C6—C1—C2—C3	2.2 (6)
O2—S1—C6—C1	−75.4 (4)	N1—C1—C2—C3	−176.5 (4)
C7—S1—C6—C1	38.5 (4)	N1—C1—C6—C5	175.2 (4)
O1—S1—C6—C5	−32.5 (4)	C2—C1—C6—S1	169.0 (3)
O2—S1—C6—C5	97.4 (4)	C2—C1—C6—C5	−3.5 (6)
C7—S1—C6—C5	−148.7 (3)	C1—C2—C3—C4	−0.1 (7)
O1—S1—C7—C8	30.5 (4)	C2—C3—C4—C5	−0.8 (7)
O2—S1—C7—C8	−99.3 (4)	C3—C4—C5—C6	−0.5 (7)
C6—S1—C7—C8	147.4 (4)	C4—C5—C6—C1	2.7 (7)
O1—S1—C7—C12	−154.8 (3)	C4—C5—C6—S1	−169.7 (4)
O2—S1—C7—C12	75.5 (4)	S1—C7—C12—C11	−172.2 (3)
C6—S1—C7—C12	−37.8 (4)	S1—C7—C12—N1	10.6 (5)
C12—N1—C13—C14	113.2 (4)	C12—C7—C8—C9	−2.4 (7)
C13—N1—C1—C2	−22.4 (6)	S1—C7—C8—C9	172.2 (4)
C12—N1—C1—C6	−24.9 (6)	C8—C7—C12—C11	2.4 (6)
C12—N1—C1—C2	153.8 (4)	C8—C7—C12—N1	−174.9 (4)
C1—N1—C13—C14	−70.6 (5)	C7—C8—C9—C10	0.3 (7)
C13—N1—C12—C7	−158.2 (4)	C8—C9—C10—C11	1.7 (7)
C13—N1—C1—C6	159.0 (4)	C9—C10—C11—C12	−1.6 (7)
C1—N1—C12—C7	25.7 (6)	C10—C11—C12—C7	−0.4 (6)
C1—N1—C12—C11	−151.4 (4)	C10—C11—C12—N1	176.8 (4)
C13—N1—C12—C11	24.8 (6)	N1—C13—C14—C15	−51.8 (6)
C18—N2—C16—C14	165.0 (4)	N1—C13—C14—C16	−170.8 (4)
C17—N2—C16—C14	−71.7 (5)	C15—C14—C16—N2	165.3 (4)
N1—C1—C6—S1	−12.3 (5)	C13—C14—C16—N2	−72.9 (5)

### Hydrogen-bond geometry (Å, °)

**Table:**

Cg2 is the centroid of the C1–C6 benzene ring.

**Table:**

*D*—H···*A*	*D*—H	H···*A*	*D*···*A*	*D*—H···*A*
N2—H2B···Cl1	0.91	2.18	3.027 (4)	155
C13—H13A···Cl1^i^	0.97	2.80	3.608 (4)	141
C13—H13B···Cl1	0.97	2.76	3.692 (4)	161
C17—H17B···Cg2^ii^	0.96	2.62	3.559 (6)	166

Symmetry codes: (i) −*x*+1, −*y*+1, −*z*+1; (ii) −*x*+1, −*y*, −*z*+1.

## References

[bb1] Amin, A. S., El-Mossalamy, M. A., Killa, H. M. & Saber, A. L. (2008). *Anal. Lett.* **41**, 80–89.

[bb2] El-Didamony, A. M. (2005). *Arch. Pharm. (Weinheim)*, **338**, 190–197. 10.1002/ardp.200400947 15864789

[bb3] Farrugia, L. J. (1997). *J. Appl. Cryst.* **30**, 565.

[bb4] Farrugia, L. J. (1999). *J. Appl. Cryst.* **32**, 837–838.

[bb5] Harrison, W. T. A., Ashok, M. A., Yathirajan, H. S. & Narayana Achar, B. (2007). *Acta Cryst.* E**63**, o3277.

[bb6] Jasinski, J. P., Pek, A. E., Nayak, P. S., Narayana, B. & Yathirajan, H. S. (2011). *Acta Cryst.* E**67**, o430–o431. 10.1107/S1600536811001851 PMC3051484 21523098

[bb7] Oxford Diffraction (2007). *CrysAlis PRO* and *CrysAlis RED* Oxford Diffraction Ltd, Abingdon, England.

[bb8] Parkin, S., Moezzi, B. & Hope, H. (1995). *J. Appl. Cryst.* **28**, 53–56.

[bb9] Sheldrick, G. M. (2008). *Acta Cryst.* A**64**, 112–122. 10.1107/S0108767307043930 18156677

[bb10] Spek, A. L. (2009). *Acta Cryst.* D**65**, 148–155. 10.1107/S090744490804362X PMC2631630 19171970

